# Effects of natural vegetative restoration on soil fungal and bacterial communities in bare patches of the southern Taihang Mountains

**DOI:** 10.1002/ece3.5564

**Published:** 2019-08-16

**Authors:** He Zhao, Xuanzhen Li, Zhiming Zhang, JianTao Yang, Yong Zhao, Zi Yang, Qili Hu

**Affiliations:** ^1^ College of Forestry Henan Agricultural University Zhengzhou China

**Keywords:** bacteria, bare patch, fungi, semi‐arid area, vegetative restoration

## Abstract

Understanding the distribution and composition of soil microbes in bare patches is a critical step to improving ecological remediation. The effects of different vegetative restoration types on soil microbes within semi‐arid bare patches remain unclear. Here, we evaluated the distribution of soil fungi and bacteria among different ecological restoration types at the southern Taihang Mountains. Analysis of variance showed that the chemical properties of soil with vegetation cover have higher nutrient quality than those of the exposed soil. The results also suggested that vegetative restoration significantly improved the diversity and the richness of the soil fungal and bacterial communities. Sequencing results showed that Ascomycota and Basidiomycota were the main soil fungal communities, whereas Proteobacteria, Acidobacteria, and Actinobacteria were the main soil bacterial communities. There were significant relationships between the contents of moisture, organic matter and organic carbon and the soil fungal/bacterial communities. Venn and network diagrams indicated that the vegetative restoration types largely influenced the soil fungi and weakly influenced the soil bacteria in the bare patches. This study discusses the importance of vegetative restoration in the ecological remediation of bare patches. These findings provide effective references for soil restorative measures, water conservation, and bare‐spot reduction at the southern Taihang Mountains in future.

## Graphical abstract

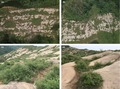

## 1 INTRODUCTION

The aggregation of soil microorganisms, plant growth, animal activity, and their living habitats forms the entire soil ecosystem (Bell, Newman, Silverman, Turner, & Lilley, 2005; Brussaard, Ruiter, & Brown, 2007). Soil microbes, as the main microcomposers of the natural ecosystem, not only participate in nutrient transport, metabolic processes, and biochemical events, but they also indirectly have a substantial direct effect on plant growth and development (e.g., mycorrhizae) (Barberán, Casamayor, & Fierer, 2014; Green & Bohannan, 2006; Tian et al., 2017). The higher the activity of the microbial community, the stronger the material cycling ability of the soil ecosystem (Rousk et al., 2010; Zuo et al., 2015). Bare patches are widespread in semi‐arid mountainous areas, and soil microbes in such areas are closely related to the vegetative cover (Singh, Dawson, Macdonald, & Buckland, 2009). Thus, an understanding of soil microbial communities in bare patches is critical for plant reconstruction and patch restoration.

What are the critical drivers of soil microbial composition and distribution? Scholars assume that spatial scales, diversity–energy relationships, temporal scales, competitive strategies, and behavior can alter soil microbial communities (Lynch et al., 2004; Prosser et al., 2007). Meanwhile, the concept of “environmental selection and adaptation” has received widespread attention in recent years (Fierer, Jackson, Vilgalys, & Jackson, 2005; Schappe et al., 2017; Tian et al., 2017). Researchers argue that the distribution of species is determined by the habitat characteristics, which is closely related to external factors (Filker, Sommaruga, Vila, & Stoeck, 2016; Lauber, Hamady, Knight, & Fierer, 2009; Leibold, 1995). Diversified vegetative restoration models, such as plantations, shrubs, forests, and grass, are different in terms of the heterogeneity of the soil spatial environment that can cause differences in soil microbial communities (Behera & Sahani, 2003; Docherty et al., 2015). Although a previous study has addressed this (Fierer, Bradford, & Jackson, 2007; Fierer, Breitbart, et al., 2007; Zhao, Li, et al., 2017; Zhao, Wang, Fan, & Song, 2017), the drivers of soil fungi and bacteria in bare patches during extremely harsh ecological conditions remain unclear.

The southern Taihang Mountains are located in a semi‐arid area. Soil erosion and degradation is prevalent in this area, which usually manifest as large areas of bare patches on the slopes, with the distribution pattern resembling a mixed mosaic of exposed soil and vegetation (Gao, Bojie, Yihe, Liu, & Wang, 2013; Zhao et al., 2018). The study of different restoration strategies can contribute to the improvement of local ecological environments (Cortina et al., 2011; Mcglone et al., 2012; Wang, Fu, Lu, & Chen, 2011). For instance, vegetative coverage can control soil erosion and reduce the diffusion of bare patches (Zhang, Xi, & Li, 2006; Zhao, Li, et al., 2017; Zhao, Wang, et al., 2017). Although soil microbes are sensitive to the adverse ecological conditions (Zhao, Li, et al., 2017; Zhao, Wang, et al., 2017), the different vegetative restoration types in bare patches are often ignored.

Using high‐throughput sequencing, soil fungi (ITS) and soil bacteria (16S rRNA) in bare patches at the southern Taihang Mountains were sequenced. We focused on two questions as follows: (a) How do the composition of fungi and bacteria in bare patches vary among the different vegetative restoration types? (b) Which environmental factors are the major drivers of fungal and bacterial distribution in the semi‐arid Mountains?

## 2 METHODS

### 2.1 Study area

**Figure 1:**
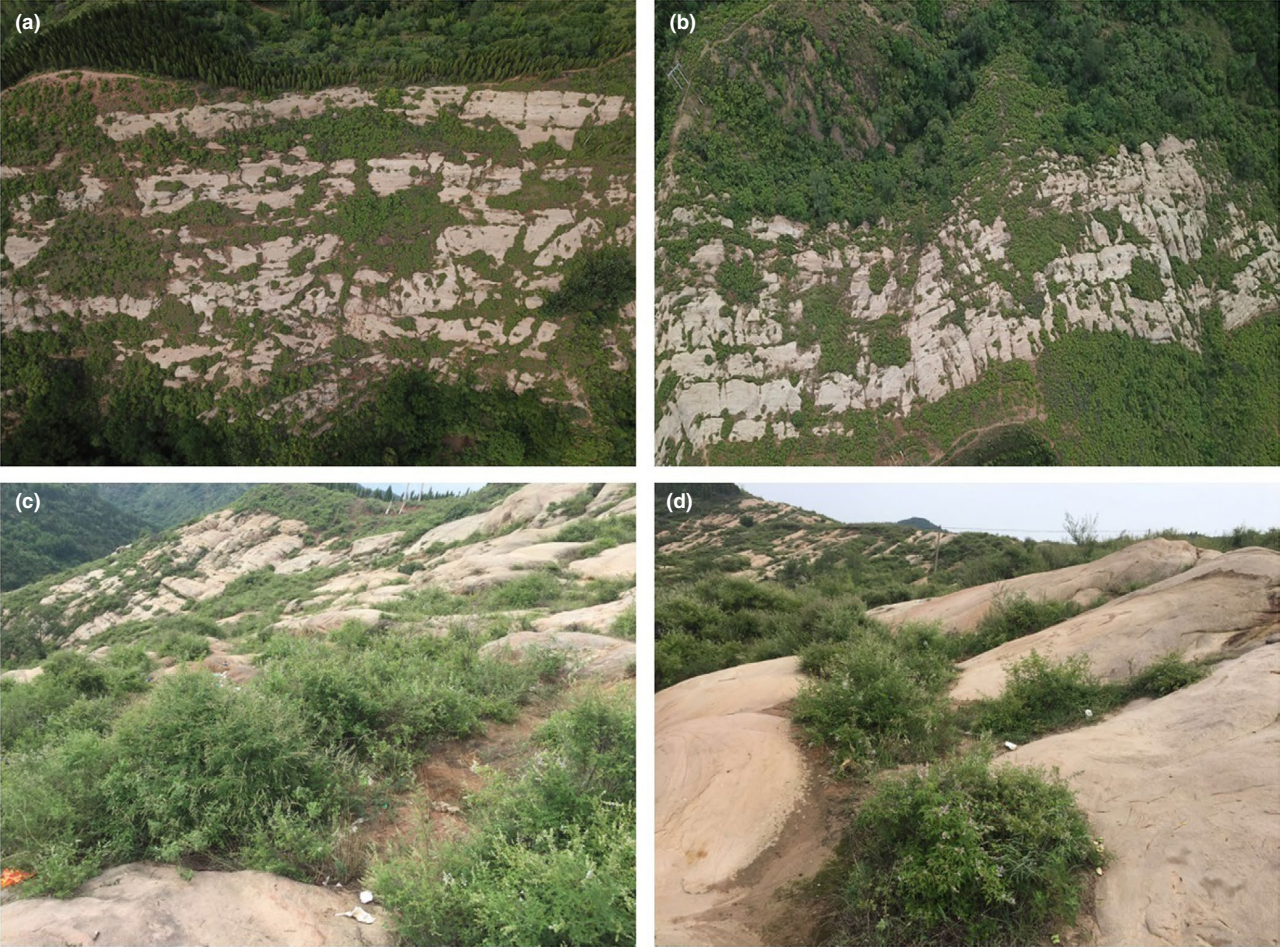
Some local harsh habitat conditions in the southern Taihang Mountains. Note: The pictures were taken by the author himself at the southern Taihang Mountains. (a) and (b) were taken by unmanned aerial vehicle; (c) and (d) were taken by a camera

The study area was located in the southern Taihang Mountains (112°28′–112°30′E, 35°01′–35°03′N), a semi‐arid region. The local climate was continental monsoon, the average air temperature was 14.3°C, the average annual sunshine rate was 54%, and the annual precipitation was 440–860 mm. The soil in the research area belonged to the Ustalf type. The vegetation in the study area experienced extensive deforestation in the 1970s, and the forest was destroyed grievously (Figure 1). Therefore, most of the bare‐patch areas are natural restoration.

### 2.2 Sample collection

Based on satellite images and unmanned aerial vehicle (UAV) detection findings, we established four bare patches of 20 m × 20 m in different latitudes and longitudes. The bare‐patch coordinates were as follows: 1 (112°29′18.24 E; 35°01′23.45 N), 2 (112°29′29.64 E; 35°01′48.66 N), 3 (112°29′12.34 E; 35°02′05.85 N), and 4 (112°29′54.31 E; 35°01′07.53 N). The standard study plot was a sunny slope that avoided rivers, valleys, roads, ridges, and forest margins. The boundary was measured with a compass. The degree of the slope was 15–35°, and the site conditions were similar across the four bare patches. Each bare patch was divided into four plots that included small trees, shrubs, grass, and exposed soil (without plant cover) of the vegetative restoration type (each plot was 10 m × 10 m). Soil samples were collected along the diagonal line using a five‐point sampling method (at a soil depth of 5–10 cm), and soil samples from each 10 m × 10 m plot were considered as an entire sample. A total of 16 soil samples were collected (4 bare patches, 4 repeats per vegetative restoration type), including four soil samples from small trees, shrubs, and grasslands, as well as the exposed soil. Small trees included mainly *Quercus variabilis Bl*. and *Robinia pseudoacacia* L. Dominant shrubs were *Vitex negundo* L. and *Lespedeza bicolor* Turcz. The dominant grasses were *Setariaviridis* (L.) Beauv and *Artemisia princeps* H. Lév. and Vaniot. After removing the soil impurities, such as stones and plant branches, 200 g of soil was collected from each vegetative restoration type. The soil samples were transported to the laboratory in an icebox and stored at −70°C until subsequent analysis. In addition, the vegetative growth in the bare patches of Taihang Mountains was relatively slow. To protect the vegetation in the bare patches, we did not collect plant tissue (roots, stems, and leaves) samples, and after collecting the soil samples, we carried out effective backfilling to reduce disruption to the environment.

### 2.3 Soil physicochemical analysis

The soil pH value and contents of moisture, available potassium, available nitrogen, and organic matter were measured following Bao (2000) (soil agricultural chemistry analytical, published by China Agricultural Press). In addition, the contents of total nitrogen and organic carbon were determined by an element analyzer (Perkin Elmer 2400; Perkin Elmer).

### 2.4 MiSeq sequencing steps

DNA was extracted from each soil sample (50 mg) using a DNA Separation Kit (Q‐BIO). The DNA was stored at −20°C for PCR detection. Agarose gel was used to detect the preliminary integrity of DNA. Concentration detection (standard 5–50 ng/µl) used Qubit 3.0 and Nanodrop for purity detection (260/280, standard 1.6–2.2). High‐throughput sequencing was used to detect fungi (ITS sequence, ITS1 region) and bacteria (16S rRNA gene fragment, V4–V5 region). The ITS sequence is conservative and consistent, and differences between species are obvious. At the same time, the smaller fragments are easy to analyze. Sequence analysis of the 16S rRNA gene fragments has been widely used in the analysis of bacterial diversity. The fungal primers used were as follows: forward, 5'–CTTGGTCATTTAGAGGAAGTAA–3' and reverse, 5'–GCTGCGTTCTTCATCGATGC–3' (Gardes & Bruns, 1993; Uroz et al., 2016). The bacterial primers used were as follows: forward, 5'–GTGCCAGCMGCCGCGGTAA–3' and reverse, 5'–CCGTCAATTCMTTTGAGTTT–3' (Rinke et al., 2014). The two‐step polymerase chain reaction (PCR) was used to construct the data library. The detailed steps of the two‐step PCR are shown in Table S1. Prior to library pooling, the barcoded PCR products were purified using a DNA Gel Extraction Kit (Axygen) and quantified using the FTC‐3000 TM Real‐Time PCR System. The libraries were sequenced by 2*300 bp paired‐end sequencing on the MiSeq platform using the MiSeq v3 Reagent Kit (Illumina) at Tiny Gene Bio‐Tech (Shanghai) Co., Ltd.

### 2.5 Bioinformatics method

The raw fastq files were de‐multiplexed based on the barcode. PE reads for soil samples were processed by Trimmomatic Software (version 0.35), and then, various parameters (slidingwindow: 50:20 minlen: 50) were used to remove the low‐quality base pairs. The trimmed reads were then further merged using the FLASH Program (version 1.2.11) with default parameters. The low‐quality contigs were removed based on the screen.seqs command using the filtering parameters as follows: maxambig = 0, minlength = 150, maxlength = 580, and maxhomop = 8. The 16S and ITS1 sequences were analyzed using Mothur Software (version 1.33.3) and UPARSE Software (version 8.1.1756). The de‐multiplexed reads were clustered at 97% sequence identity into operational taxonomic units (OTUs) using the UPARSE pipeline. The OTU representative sequences were assigned for taxonomy against Silva 128 database for 16S and UNITE for ITS with a confidence score ≥0.6 by the classify.seqs command in Mothur Software.

### 2.6 Statistical procedures

The OTU indices of Chao, richness, Good's coverage, and Shannon were analyzed by Mothur Software (version 1.33.3), following the method of Schloss et al. (2009). One‐way analysis of variance was performed with SPSS Software (version 19.0). The boxplot was prepared using the “boxplot” library. The diagram of shared OTUs was generated with the “VennDiagram” library. Cytoscape Software (version 2.8) was used to produce a diagram of the network, following the method of Smoot, Ono, Ruscheinski, Wang, and Ideker, (2011). The redundancy analysis and “envfit” function were graphed using the “Vegan” library in R language Software (version 3.4.4) (Oksanen, Kindt, & Legendre, 2007), and the diagrams were optimized by Canoco Software (Windows 4.5 package; Braak & Smilauer, 1998). Both fungal and bacterial sequences were deposited into the NCBI database, with accession numbers PRJNA522045 and PRJNA522071, respectively.

## 3 RESULTS

### 3.1 Overview of soil fungal and bacterial data

The chemical properties of soil varied with the vegetative restoration type (Table 1). The soil in the bare patches of Taihang Mountains had a pH value ranging from 8.18 to 8.48. The available potassium in the soil ranged from 12.22 to 14.33 mg/kg, organic matter ranged from 17.3 to 26.39 g/kg, available nitrogen ranged from 116.83 to 170.58 mg/kg, total nitrogen ranged from 0.14% to 0.44%, and organic carbon ranged from 2.19% to 3.16%. According to Chao and Shannon indices, the richness of the fungal communities ranged from 300.4 to 540.4, and the diversity ranged from 2.7 to 4.6. The richness of the bacterial communities ranged from 1,095.71 to 2069.70, and the diversity ranged from 5.22 to 5.93.

**Table 1:** Overview of soil fungal and bacterial sequence data and chemical properties

Variables	Minimum	Average value	Maximum
pH	7.16	7.21 ± 0.05	7.30
Moisture content (%)	14.35	15.91 ± 1.21	17.03
Available potassium (mg/kg)	12.22	13.06 ± 1.06	14.33
Soil organic matter (g/kg)	1,730	21.72 ± 1.19	26.39
Available nitrogen (mg/kg)	116.83	144.49 ± 3.94	170.58
Total nitrogen (%)	0.14	0.36 ± 0.03	0.44
Soil organic carbon (%)	2.19	2.65 ± 0.23	3.16
Fungal Chao1 (richness)	300.44	427.20 ± 41.92	540.24
Fungal Shannon (diversity)	2.73	3.74 ± 0.18	4.59
Fungal coverage (%)	0.98	0.99 ± 0.001	0.99
Bacterial Chao1 (richness)	1,095.71	2004.94 ± 94.33	2069.70
Bacterial Shannon (diversity)	5.22	5.69 ± 0.07	5.93
Bacterial coverage (%)	0.98	0.98 ± 0.001	0.99

### 3.2 Soil data analyses of the different vegetation restoration types in bare patches

Variance analysis showed that both richness and diversity (fungi and bacteria) were significantly higher in vegetative restoration (small trees, shrub, and grass) than that in exposed soil (Table 2). Among the different vegetative restoration types, small trees had the highest values of richness and diversity.

**Table 2:** OTU data of soil fungi and bacteria among the different vegetative restoration types

Types	Soil fungi			Soil bacteria		
	Coverage (%)	Chao (richness)	Shannon (diversity)	Coverage (%)	Chao (richness)	Shannon (diversity)
Small tree	0.9992 ± 0.0002 A	540.2415 ± 58.0069 A	4.5915 ± 0.1969 A	0.9815 ± 0.0003 A	2,269.0133 ± 38.1182 A	5.9365 ± 0.0315 A
Shrub	0.9992 ± 0.0001 A	422.9109 ± 49.0241 AB	3.9504 ± 0.2698 B	0.9863 ± 0.0012 A	2069.6987 ± 69.5010 A	5.8902 ± 0.0643 A
Grass	0.9990 ± 0.0003 A	445.2072 ± 28.7213 A	3.6903 ± 0.0395 B	0.9833 ± 0.0010 A	2090.3611 ± 46.7138 A	5.7306 ± 0.0128 A
Exposed soil	0.9993 ± 0.0002 A	300.4473 ± 31.9431 B	2.7330 ± 0.2039 C	0.9835 ± 0.0038 A	1,590.7053 ± 223.0134 B	5.2216 ± 0.1593 B

Note

One‐way analysis of variance (ANOVA) was used to evaluate the statistical significance and results, followed by Tukey's HSD test. Capital direction symbols indicate full (5%) significance.

The analysis of geochemical characteristics showed that small trees had the highest content of available nitrogen in the soil, reaching 170.58 mg/kg (values quoted are averages, the same below), and both organic matter and organic carbon levels in the soil of small trees were significantly higher than those in the exposed soil, reaching 26.39 g/kg and 3.16%, respectively (Table 3). The total nitrogen content in the soil of small trees, shrubs, and grass was significantly higher than that in exposed soil. The results confirmed that soil nutrients and conditions can be improved by plant cover, and vegetative restoration may play an active role in the rehabilitation of bare patches.

**Table 3:** Geochemical characteristics of the different vegetative restoration types

Types	pH	Moisture content (%)	Available potassium (mg/kg)	Soil organic matter (g/kg)	Available nitrogen (mg/kg)	Total nitrogen (%)	Soil organic carbon (%)
Small tree	7.16 ± 0.04 A	16.85 ± 1.40 A	14.34 ± 1.39 A	26.39 ± 1.53 A	170.58 ± 1.18 A	0.44 ± 0.02 A	3.16 ± 0.19 A
Shrub	7.20 ± 0.04 A	17.03 ± 0.69 A	12.15 ± 1.11 A	22.61 ± 0.38 B	155.85 ± 3.78 B	0.44 ± 0.02 A	2.88 ± 0.19 AB
Grass	7.22 ± 0.06 A	15.41 ± 1.69 A	13.52 ± 0.60 A	20.59 ± 0.85 BC	134.73 ± 5.14 C	0.41 ± 0.06 A	2.39 ± 0.20 AB
Exposed soil	7.30 ± 0.07 A	14.35 ± 1.07 A	12.22 ± 1.13 A	17.30 ± 2.01 C	116.83 ± 5.66 C	0.14 ± 0.03 B	2.19 ± 0.35 B

Note

Capital direction symbols indicate full (5%) significance.

### 3.3 Soil fungal and bacterial composition in bare patches in the southern Taihang Mountains

**Figure 2:**
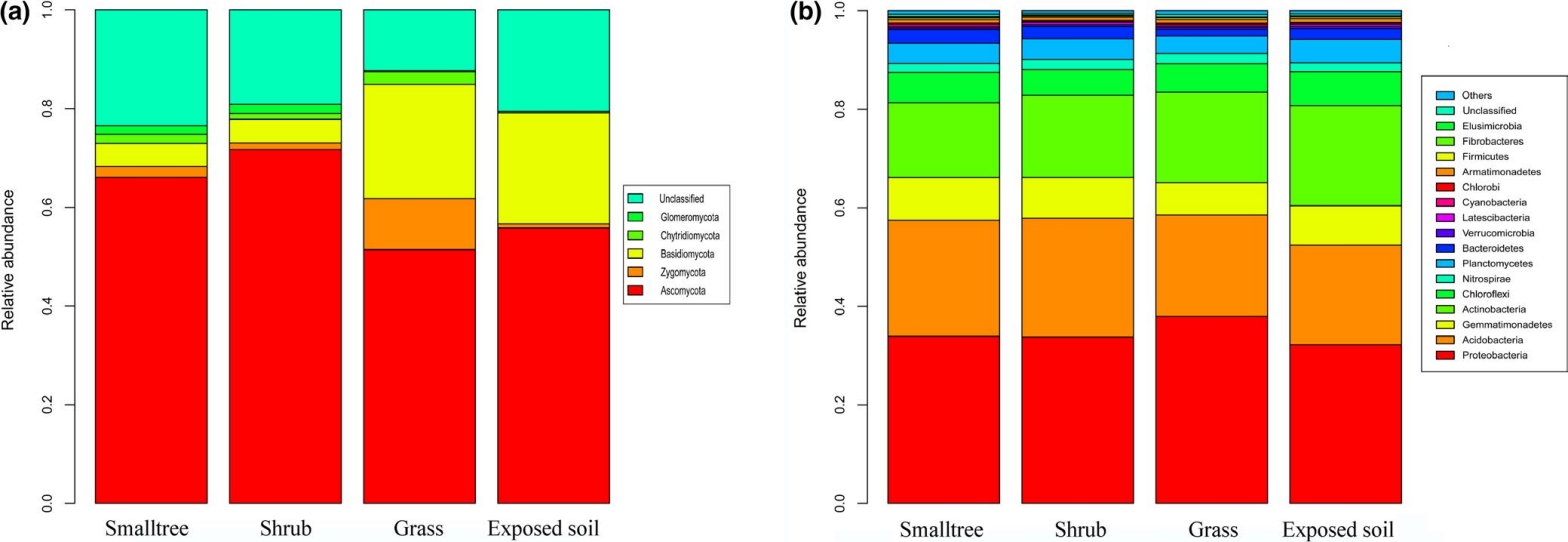
Relative abundances of the soil fungal and bacterial microbes among the different vegetative restoration types in the bare patches (phylum level). (line 169). Note: (a) fungi; (b) bacteria

Soil fungal and bacterial composition differed among the vegetative restoration types in bare patches. Soil fungal communities consisted mainly of Ascomycota, Basidiomycota, Chytridiomycota, Zygomycota, and Glomeromycota, and sequences that could not be identified were considered unclassified (Figure 2). Within the fungal communities, Ascomycota and Basidiomycota were the dominant soil fungal groups, and they accounted for 57%–81% of the total fungal composition. A study of the relative abundance of bacterial microbes in the soil showed that Proteobacteria, Acidobacteria, and Actinobacteria were the dominant bacteria in the bare patches, accounting for 63%–78% of the total bacterial composition (Figure 2). Compared with the distribution of fungi in the soil, the distribution of bacteria was more uniform, indicating that the type of vegetative restoration influenced the distribution of vegetation. However, changes in the vegetative restoration type had less impact on the bacterial communities. The detailed data of the soil fungal and bacterial phylum are shown in Table S2.

### 3.4 Differences and similarities in the soil fungal and bacterial OTUs among the different vegetative restoration types

**Figure 3:**
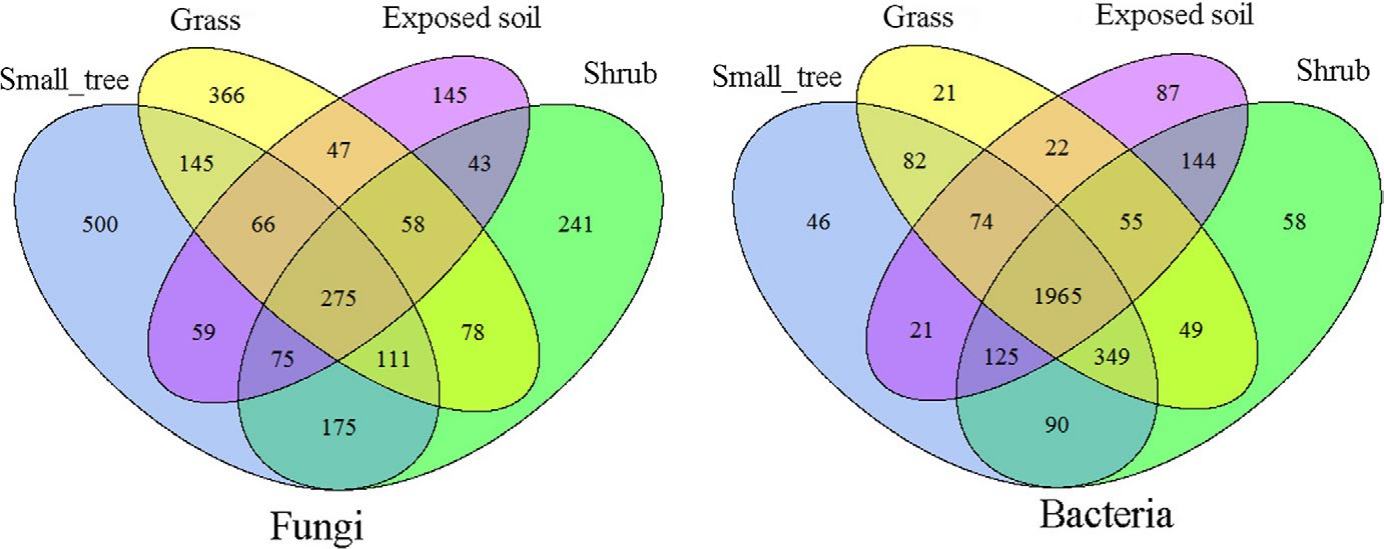
Venn diagrams of the fungal and bacterial OTUs among different vegetative restoration types in the bare patches. (line 183)

Venn diagrams revealed that the distribution of OTUs of the fungal and bacterial communities in the four vegetative restoration types was different (Figure 3). In fungal communities, the minimum number of OTUs (766) was found in the exposed soil, and the maximum number of OTUs (1,405) was found in small trees. The highest number of OTUs (500) existed in small trees, and the lowest number of OTUs (145) existed in the exposed soil. Furthermore, there were 275 common OTUs across the four vegetative restoration types, accounting for approximately 11.5% of the total number of OTUs. In bacterial communities, the minimum number of OTUs (2,491) was found in the exposed soil, and the maximum number of OTUs (2,834) was found in shrubs. Compared with the total number of OTUs in fungi, there were 1965 common OTUs of bacteria across the four vegetative restoration types, accounting for 61.7% of the total number of OTUs.

**Figure 4:**
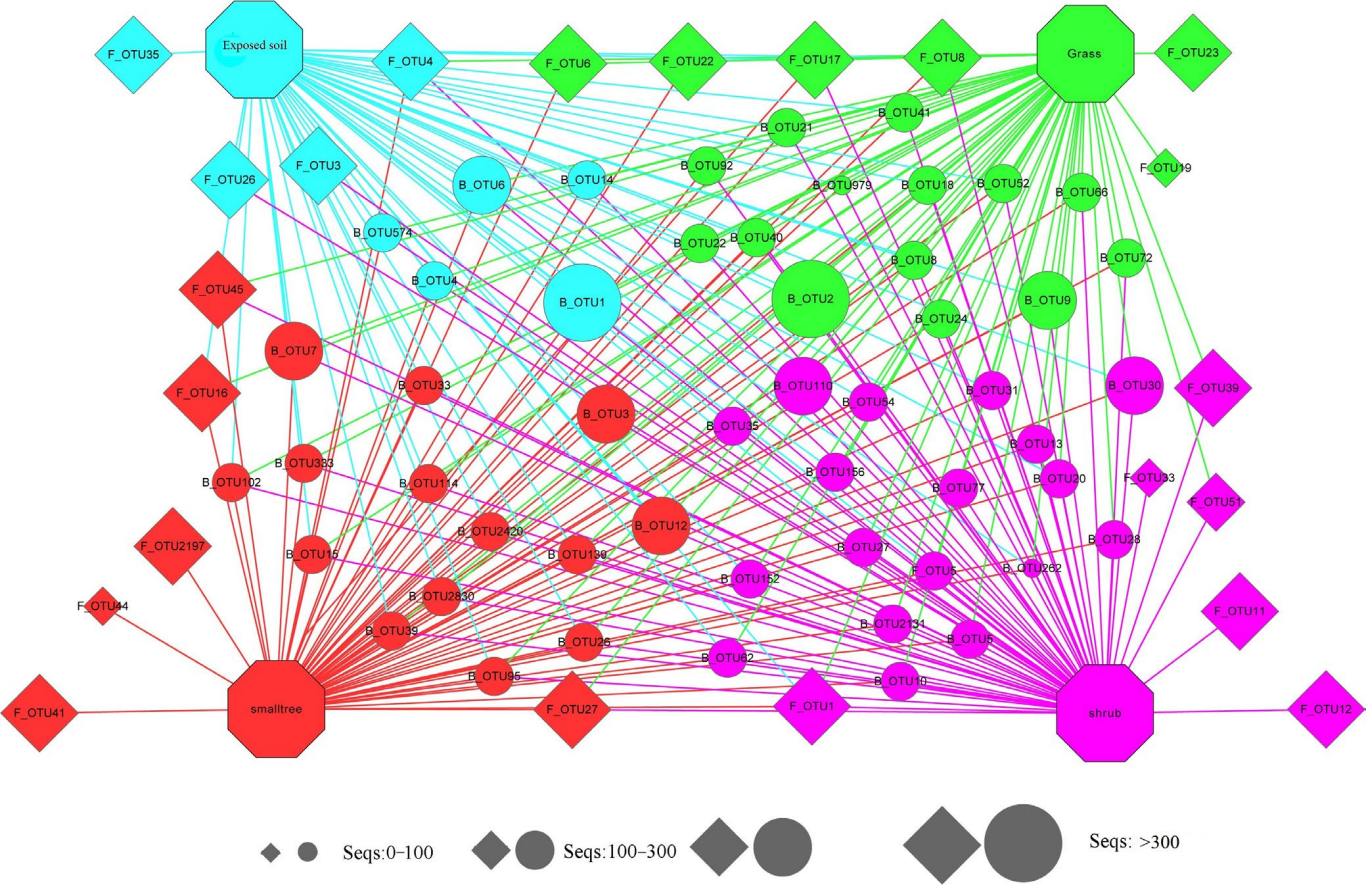
Network of the top 50 fungal and bacterial OTUs in the bare‐patch area. (line 194). Note: Rhombus represents the fungal OTUs; circle represents the bacterial OTUs. The octagon of different colors represents the vegetative restoration type, whereas the circular nodes represent the OTUs that connect to the different vegetative restoration types through edges (lines). The center color of the circular node represents the specific soil vegetative restoration type that had the highest number of sequenced OTUs among the four vegetative restoration types

The network diagrams revealed that the soil microbial (top 50 fungal and bacterial OTUs) mainly appeared in the vegetative restoration types (Figure 4). In general, F_OTU_1 (Ascomycota) was the dominant fungal community, and B_OTU_1 (Proteobacteria) and B_OTU_2 (Acidobacteria) were the dominant bacterial communities. The sequence numbers 100–300 were the dominant OTUs in the different vegetative restoration types. Furthermore, the diagram showed that fungal communities with a higher sequence mainly existed within a single vegetative type.

### 3.5 Effect of environmental factors on the soil fungal and bacterial communities in bare patches

**Figure 5:**
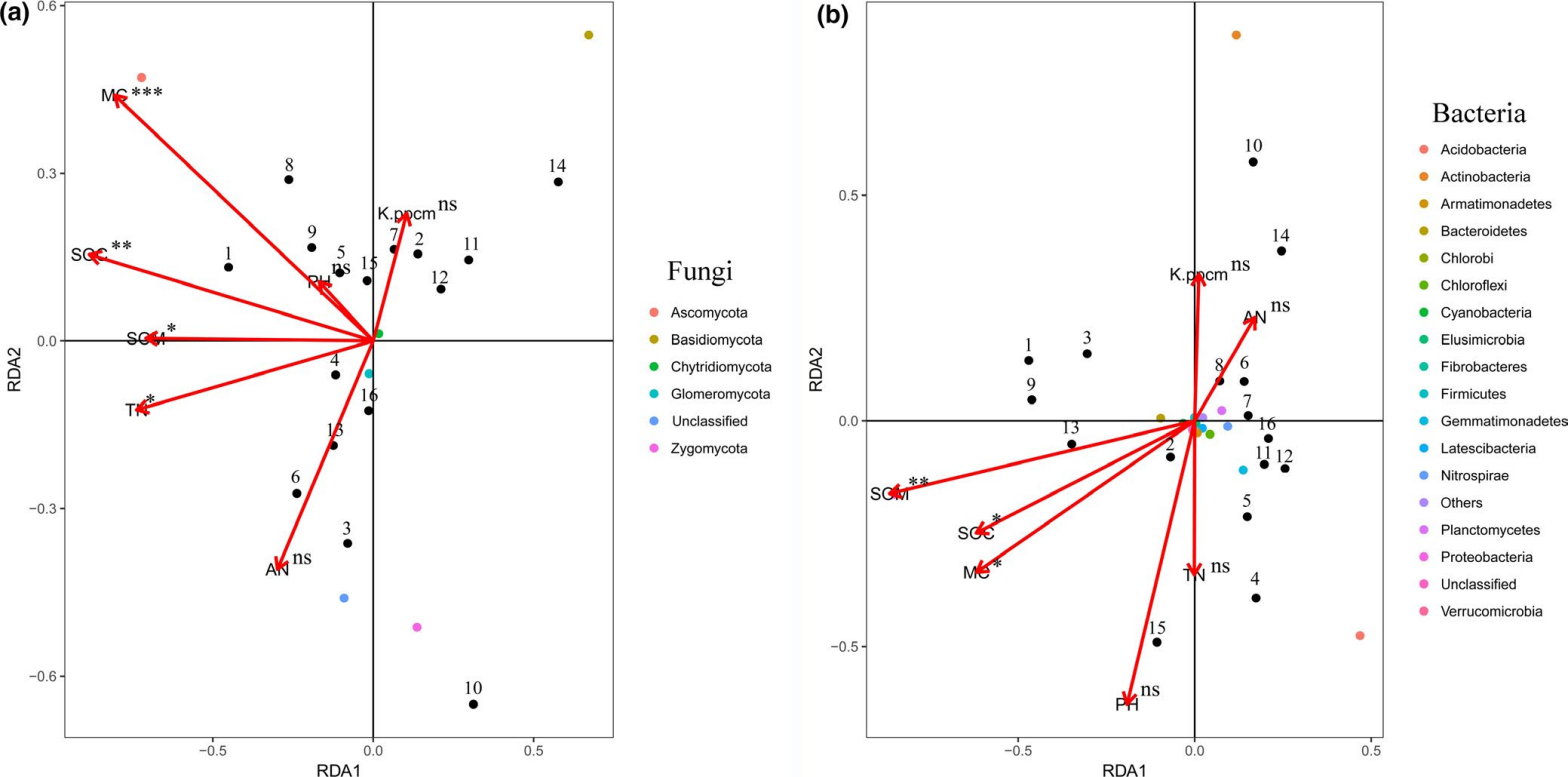
Distance‐based redundancy tests used to interpret the correlations between the soil microbes and environmental properties. (line 202). Note: (a) fungi, (b) bacteria; MC, moisture content; K ppcm, available potassium; SOM, soil organic matter; AN, available nitrogen; TN, total nitrogen; SOC, soil organic carbon. Number represents sample, 1 ~ 4: Small tree; 5 ~ 8: Shrub; 9 ~ 12: Grass; 13 ~ 16: Exposed soil. *Correlations were significant at the .05 level. **Correlations were significant at the .01 level. *p*‐values based on 999 permutations

The diagrams of all vegetative restoration types and soil microbe communities revealed that the soil microbial communities in the bare patches were closely related to external factors, and various environmental factors that affected the driving ability of microbes differently. The Monte Carlo test showed that the moisture content (*r* ^2^ = .719, *p* = .001), soil organic carbon content (*r* ^2^ = .665, *p* = .002), soil organic matter content (*r* ^2^ = .406, *p* = .038), and total nitrogen content (*r* ^2^ = .444, *p* = .014) were the main factors affecting the distribution of fungal communities in the soil (Figure 5a, Table S3). On the other hand, the soil organic matter content (*r* ^2^ = .732, *p* = .002), moisture content (*r* ^2^ = .415, *p* = .03), and organic carbon content (*r* ^2^ = .396, *p* = .045) were the main factors influencing the distribution of bacterial communities in the soil (Figure 5b, Table S3). However, the effects of the pH, available potassium, and available nitrogen in the soil were less influential on fungal and bacterial communities in bare patches.

## 4 DISCUSSION

Our study showed that the soil chemical properties of the vegetative restoration types were better than those of the exposed soil. Compared with other restoration types, the contents of available nitrogen, soil organic matter, and organic carbon in small trees were significantly higher than those in exposed soil. Vegetative cover can enrich the soil with nutrients, such as litter and particulate matter accumulation (Burton, Pregitzer, & Hendrick, 2000; Corstanje, Reddy, Prenger, Newman, & Ogram, 2007; Raiesi & Beheshti, 2014). Small trees were present in the system much longer than the other types, and they had a larger canopy width and litter acquisition ability; thus, its soil chemical properties were better than those of the other restoration types (Gordon & Jackson, 2000; Liu, Fu, Zheng, & Liu, 2010). Our results confirm that vegetative restoration can improve soil conditions and play an active role in the restoration of bare patches.

Variance analysis showed that the fungal/bacterial diversity and richness of the vegetative restoration soil were significantly greater than those of the exposed soil. This phenomenon may have been due to the fact that the vegetation‐covered habitat provided stable shelter and adequate sources of nutrition for soil microbes (Corstanje et al., 2007) and improved habitat conditions that are conducive to the multiplication and diffusion of soil microbes, resulting in a large number of microbial communities in the vegetatively restored soil (Jing, Cheng, Jin, Su, & Yu, 2014). Our results confirmed that vegetative restoration could promote the diffusion and distribution of soil microbial communities. It was also found that the soil restored by small trees had the largest diversity of fungi and bacteria. The harsh habitat conditions in bare patches limited the spread of soil microbial communities (Long et al., 2010), and these adverse conditions in small trees could be alleviated more than in other vegetative types (Burton et al., 2000; Gordon & Jackson, 2000). The results indicate that vegetative restoration with complete habitats (abundant nutrients and improved habitats) is beneficial to the reproduction and distribution of microbial communities.

Despite the harsh ecological environment and the challenging survival conditions in the bare patches of Taihang Mountains (Zhao, 2007), we were able to obtain relatively integrated soil fungal and bacterial community information (Tables S4 and S5). The results showed that Ascomycota and Basidiomycota were the main fungal communities in the bare patches. Researchers have argued that Ascomycota and Basidiomycota communities can develop the ability to adapt to conditions of drought during growth and evolution (Bjorbækmo et al., 2010; Leff et al., 2015; Treseder et al.., 2014). For the bacterial communities, Proteobacteria, Acidobacteria, and Actinobacteria were the main bacterial phyla in the bare patches, accounting for approximately 63%–78% of the total community. These findings were similar to those reported previously (Fierer, Bradford, et al., 2007; Fierer, Breitbart, et al., 2007; Tian et al., 2017). Meanwhile, Proteobacteria was the dominant community, possibly because most Proteobacteria communities have both autotrophic and heterotrophic forms, as well as phototrophic and chemotrophic forms. Thus, these abilities enable Proteobacteria communities to survive and reproduce in adverse conditions (Campbell, Annette Summers, Porter, & Ken, 2006; Moulin, Munive, Dreyfus, & Boivin‐Masson, 2001).

In contrast to the study of Arctic vegetation types (Chu, Neufeld, Walker, & Grogan, 2011), our study yielded opposite results on fungi and bacteria. The Venn chart showed that the total number of bacterial OTUs was 61.7% and that of fungi was 11.5%, indicating that bacterial communities are widely distributed in the bare patches. The network map also confirmed that the dominant soil fungal communities mainly existed in a single vegetative cover type, whereas bacterial communities appeared in all vegetative restoration types. Researchers contend that volumes for bacterial communities are usually smaller than those for fungal communities, so more copies can be obtained during sequencing (Bailly et al., 2007). Our investigation of these soil microbial groups, which were obtained from various bare patches, indicates that different vegetative restoration types have significant influence on the distribution of the major soil fungi, and relatively little influence on the distribution of the major soil bacteria.

The composition of microbial communities is usually influenced by the habitat type and the chemical properties of the soil (Zhao, Li, et al., 2017; Zhao, Wang, et al., 2017). The environmental driving factors on soil microbes have been widely recognized (Lennon, Aanderud, Lehmkuhl, & Schoolmaster, 2012). The db‐RDA indicated that various environmental factors have different effects on the distribution of fungal and bacterial communities in the soil. The results showed that the contents of moisture, soil organic matter, and organic carbon were closely related to soil fungal and bacterial communities, whereas the total nitrogen content could significantly drive the distribution of soil fungal communities only. Adverse conditions of water shortage are prevalent in bare patches. Therefore, the moisture content is an important factor for the survival of microbes (Falconer, Houston, Otten, & Baveye, 2012; Idowu, Edema, & Adeyi, 2006; Postma, Veen, & Walter, 1989), and the moisture content directly determines the reproductive or metabolic processes of soil microbes (Gray, 1985; Skopp, Jawson, & Doran, 1990). Furthermore, the chemical properties of soil can enhance the nutrition of soil microbes, and a lack of soil nutrients can adversely affect the reproduction and diffusion of microbial communities (Zaller, Frank, & Drapela, 2011; Zhang, Liu, Xue, & Wang, 2016). Organic carbon, as a main component of soil matter, is a critical source of nutrients for microbial communities. In the presence of organic carbon, microbes can maintain a high growth rate (Fierer, Bradford, et al., 2007; Fierer, Breitbart, et al., 2007). In general, our research considered the relationship between restoration variables or soil chemical properties and soil microbes in bare patches. However, further studies on the allocation of the various vegetative types to achieve the best restoration effect are needed in future.

## 5 CONCLUSION

Understanding the distribution and the composition of soil microbes will provide new insights on bare‐spot control and ecological restoration at semi‐arid mountainous area. Our results confirmed the following: (a) The chemical properties of soil in vegetative restoration soil were better than those of the exposed soil, indicating that vegetation‐covered soil could play a positive role in the restoration of bare patches. (b) The diversity and richness of the vegetative restoration type soil were significantly higher than those of exposed soil. Ascomycota and Basidiomycota were the dominant communities of fungi in the soil, and Proteobacteria, Acidobacteria, and Actinobacteria were the dominant communities of bacteria in the bare patches. (c) The contents of moisture, soil organic matter, and organic carbon were important driving factors of the distribution of fungal and bacterial communities. (d) Lastly, the vegetative restoration types had a stronger influence on the soil fungal than the bacterial communities in the semi‐arid mountains. Our results provide new insights on how to improve vegetative restoration in bare patches.

## CONFLICT OF INTEREST

None declared.

## AUTHOR CONTRIBUTIONS

Yong Zhao designed the experiments. He Zhao conducted the experiments, analyzed most of the results, and wrote the paper. Xuanzhen Li and Zhiming Zhang revised this paper. Jiantao Yang, Zi Yang, and Qili Hu performed the data analysis. All authors have approved the final manuscript.

## Data Availability

Both fungal and bacterial sequence data for this paper have been archived into the NCBI database. The accession numbers were PRJNA522045 and PRJNA522071. And the sequence information has been released.
