# Supplementary material for: Effects of natural vegetative restoration on soil fungal and bacterial communities in bare patches of the southern Taihang Mountains
Source: Ecol Evol. 2019 Aug 16;9(18):10432–41. doi: 10.1002/ece3.5564 (PMC6787810; doi:10.1002/ece3.5564)
Supplement: Supplementary file 1 [file ECE3-9-10432-s001.docx]

Appendix S1

Figures


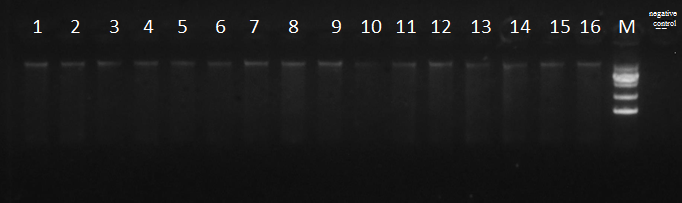


Fig. S1. Electrophoresis Map of Genome Extraction


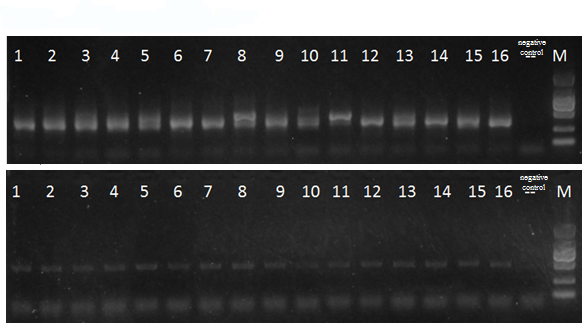


Fig. S2. PCR amplified electrophoresis

Note: The figure above was ITS, and the figure below was 16s.

Tables:

Table S1 More detailed steps of the two-step PCR.

|  | PCR step | | | | | | |
| --- | --- | --- | --- | --- | --- | --- | --- |
|  | 5xBuffer | dNTP (10mM) | DNA polymerase | F/R Inner primer(10 uM) | Template | ddH2O | |
| First PCR | 10μL | 1μL | 1U | Each 1μL | 5ng-50ng | fill up to 50 μL | |
| Second PCR | 8μL | 1μL | 0.8 U | Each 1μL | 5uL | fill up to e0 μL | |
|  | PCR system | | | | | | |
| First PCR | 35 cycles | 94℃ | 94℃ | 55℃ | 72℃ | 72℃ | 10℃ |
|  |  | 2min | 35s | 35s | 30s | 5min | heat preservation |
| Second PCR | 11 cycles | 94℃ | 94℃ | 56℃ | 72℃ | 72℃ | 10℃ |
|  |  | 2min | 35s | 35s | 30s | 3min | heat preservation |

Note: PCR instrument(Applied Biosystems 9700 , USA). F/R Inner primer:

Table S2 More detailed date of the soil fungal and bacterial phylum

| types | Smalltree | | | Shrub | | | Grass | | | Exposed soil | | |
| --- | --- | --- | --- | --- | --- | --- | --- | --- | --- | --- | --- | --- |
| fungi | average value |  | standard error | average value |  | standard error | average value |  | standard error | average value |  | standard error |
| Ascomycota | 0.6606 | a | 0.0798 | 0.7172 | a | 0.0570 | 0.5144 | a | 0.087 | 0.5582 | a | 0.1168 |
| Zygomycota | 0.0226 | a | 0.0060 | 0.0134 | a | 0.0018 | 0.1032 | a | 0.081 | 0.0081 | a | 0.0013 |
| Basidiomycota | 0.0466 | a | 0.0129 | 0.0478 | a | 0.0115 | 0.2317 | a | 0.0771 | 0.2248 | a | 0.1432 |
| Chytridiomycota | 0.0181 | a | 0.0071 | 0.0114 | a | 0.0097 | 0.0253 | a | 0.0206 | 0.0004 | a | 0.0003 |
| Glomeromycota | 0.0177 | a | 0.0075 | 0.0190 | a | 0.0148 | 0.0023 | a | 0.0013 | 0.0030 | a | 0.0008 |
| unclassified | 0.2345 | a | 0.0698 | 0.1911 | a | 0.0429 | 0.1232 | a | 0.0238 | 0.2055 | a | 0.0436 |
| bacteria |  |  |  |  |  |  |  |  |  |  |  |  |
| Proteobacteria | 0.3392 | a | 0.0344 | 0.3222 | a | 0.0160 | 0.3798 | a | 0.0382 | 0.3375 | a | 0.0294 |
| Acidobacteria | 0.2353 | a | 0.0210 | 0.2019 | a | 0.0085 | 0.2412 | a | 0.0287 | 0.2056 | a | 0.0370 |
| Gemmatimonadetes | 0.0867 | a | 0.0085 | 0.0801 | ab | 0.0038 | 0.0827 | ab | 0.006 | 0.0652 | b | 0.0052 |
| Actinobacteria | 0.1521 | a | 0.0154 | 0.2033 | a | 0.0141 | 0.1667 | a | 0.0287 | 0.1840 | a | 0.0340 |
| Chloroflexi | 0.0612 | a | 0.0050 | 0.0686 | a | 0.0077 | 0.0522 | a | 0.005 | 0.0579 | a | 0.0033 |
| Nitrospirae | 0.0184 | a | 0.0023 | 0.0183 | a | 0.0036 | 0.0210 | a | 0.006 | 0.0205 | a | 0.0074 |
| Planctomycetes | 0.0410 | a | 0.0028 | 0.0475 | a | 0.0082 | 0.0418 | a | 0.0034 | 0.0360 | a | 0.0044 |
| Bacteroidetes | 0.0278 | a | 0.0075 | 0.0217 | a | 0.0029 | 0.0252 | a | 0.0078 | 0.0132 | a | 0.0030 |
| Verrucomicrobia | 0.0033 | a | 0.0004 | 0.0043 | a | 0.0008 | 0.0036 | a | 0.0005 | 0.0040 | a | 0.0009 |
| Latescibacteria | 0.0028 | a | 0.0008 | 0.0041 | a | 0.0019 | 0.0046 | a | 0.0032 | 0.0026 | a | 0.0012 |
| Cyanobacteria | 0.0044 | a | 0.0024 | 0.0026 | a | 0.0008 | 0.0015 | a | 0.0008 | 0.0035 | a | 0.0016 |
| Chlorobi | 0.0028 | a | 0.0003 | 0.0017 | a | 0.0004 | 0.0024 | a | 0.0004 | 0.0025 | a | 0.0016 |
| Armatimonadetes | 0.0070 | a | 0.0008 | 0.0078 | a | 0.0008 | 0.0068 | a | 0.0005 | 0.0075 | a | 0.0009 |
| Firmicutes | 0.0033 | a | 0.0004 | 0.0034 | a | 0.0002 | 0.0023 | a | 0.0004 | 0.0034 | a | 0.0006 |
| Fibrobacteres | 0.0009 | a | 0.0003 | 0.0006 | a | 0.0002 | 0.0006 | a | 0.0002 | 0.0003 | a | 0.0001 |
| Elusimicrobia | 0.0023 | a | 0.0005 | 0.0017 | a | 0.0003 | 0.0017 | a | a | 0.0016 | a | 0.0004 |
| unclassified | 0.0045 | a | 0.0007 | 0.0039 | a | 0.0008 | 0.0033 | a | 0.0003 | 0.0050 | a | 0.0012 |
| others | 0.0069 | a | 0.0011 | 0.0063 | a | 0.0009 | 0.0048 | a | 0.0009 | 0.0074 | a | 0.0020 |

Note: Using the one-way analysis of variance (ANOVA) to evaluate statistical significance and results, followed by Tukey’s HSD test. Lowercase letter symbols indicate full (5%) significance.

Table S3. Monte Carlo Tests were used to detect the relationship between soil microbes and environmental factor variables.

|  | RDA1 | RDA2 | r^2^ | P-value |
| --- | --- | --- | --- | --- |
| Fungi |  |  |  |  |
| pH | -0.8264 | 0.5632 | 0.034 | 0.807 |
| Moisture content (%) | -0.8574 | 0.5147 | 0.719 | 0.001*** |
| Available potassium (mg kg^−1^) | 0.3708 | 0.9287 | 0.050 | 0.706 |
| Soil organic matter (g kg^−1^) | -0.9980 | 0.0630 | 0.406 | 0.038 * |
| Available nitrogen (mg kg^−1^) | -0.5623 | -0.8269 | 0.200 | 0.246 |
| Total nitrogen (^%^) | -0.9932 | -0.1164 | 0.444 | 0.014 * |
| Soil organic carbon (%) | -0.9736 | 0.2282 | 0.665 | 0.002 ** |
|  |  |  |  |  |
| Bacteria |  |  |  |  |
| pH | -0.7602 | -0.6497 | 0.155 | 0.333 |
| Moisture content (%) | -0.9803 | -0.1973 | 0.415 | 0.030 * |
| Available potassium (mg kg^−1^) | 0.2863 | 0.9581 | 0.030 | 0.853 |
| Soil organic matter (g kg^−1^) | -0.9947 | -0.1026 | 0.732 | 0.002 ** |
| Available nitrogen (mg kg^−1^) | 0.9251 | 0.3798 | 0.046 | 0.713 |
| Total nitrogen (^%^) | -0.1828 | -0.9832 | 0.032 | 0.797 |
| Soil organic carbon (%) | -0.9870 | -0.1606 | 0.396 | 0.045 * |

Note: *Correlation is significant at the 0.05 level. **Correlation is significant at the 0.01 level. *P*-values based on 999 permutations.

Table S4. The information about top 50 OTUs soil Fungi in soil samples.

| OTUId | Small tree | | | | Shrub | | | | Grass | | | | Bare patch | | | | total | Taxonomy |
| --- | --- | --- | --- | --- | --- | --- | --- | --- | --- | --- | --- | --- | --- | --- | --- | --- | --- | --- |
|  | Plot 1 | Plot 2 | Plot 3 | Plot 4 | Plot 1 | Plot 2 | Plot 3 | Plot 4 | Plot 1 | Plot 2 | Plot 3 | Plot 4 | Plot 1 | Plot 2 | Plot 3 | Plot 4 |  |  |
| OTU1 | 494 | 1952 | 1271 | 4024 | 8154 | 2161 | 1382 | 1769 | 620 | 2102 | 1013 | 1223 | 1963 | 1360 | 1248 | 1428 | 32164 | k__Fungi(100);p__Ascomycota(100);c__Sordariomycetes(100);o__Hypocreomycetidae_ord_Incertae_sedis(100);f__Plectosphaerellaceae(100);g__Verticillium(100); |
| OTU3 | 0 | 50 | 0 | 1309 | 1 | 25 | 3666 | 1014 | 308 | 42 | 0 | 492 | 20610 | 119 | 161 | 2 | 27799 | k__Fungi(100);p__Ascomycota(93); |
| OTU2 | 1 | 3 | 0 | 193 | 1 | 0 | 0 | 685 | 2745 | 0 | 276 | 157 | 6 | 342 | 21881 | 0 | 26290 | k__Fungi(100);p__Ascomycota(95);c__Lecanoromycetes(78);o__Teloschistales(75); |
| OTU10 | 1 | 0 | 0 | 1 | 15 | 0 | 122 | 1 | 0 | 0 | 0 | 10 | 2 | 24775 | 0 | 1 | 24928 | k__Fungi(100);p__Basidiomycota(100);c__Agaricomycetes(100);o__Agaricales(100);f__Agaricaceae(100);g__Leucoagaricus(100);s__Leucoagaricus_purpureolilacinus(60); |
| OTU4 | 396 | 113 | 2127 | 2103 | 3 | 496 | 165 | 130 | 615 | 333 | 58 | 1578 | 97 | 194 | 58 | 6801 | 15267 | k__Fungi(100);p__Ascomycota(100);c__Sordariomycetes(100);o__Hypocreales(100);f__Nectriaceae(100);g__Fusarium(92); |
| OTU21 | 0 | 0 | 0 | 1 | 8 | 0 | 0 | 3 | 23 | 0 | 0 | 14062 | 0 | 7 | 0 | 0 | 14104 | k__Fungi(100);p__Basidiomycota(100);c__Agaricomycetes(100);o__Agaricales(100);f__Agaricaceae(99);g__Leucoagaricus(98);s__Leucoagaricus_orientiflavus(75); |
| OTU11 | 0 | 20 | 0 | 63 | 2 | 2 | 3705 | 7720 | 42 | 0 | 77 | 936 | 0 | 55 | 657 | 0 | 13279 | k__Fungi(100);p__Ascomycota(83); |
| OTU9 | 0 | 22 | 1 | 283 | 0 | 0 | 2424 | 8563 | 138 | 0 | 17 | 50 | 4 | 590 | 57 | 0 | 12149 | k__Fungi(100);p__Ascomycota(100); |
| OTU5 | 303 | 2038 | 1483 | 1486 | 61 | 930 | 489 | 487 | 363 | 1810 | 159 | 609 | 346 | 225 | 512 | 782 | 12083 | k__Fungi(100);p__Ascomycota(100);c__Sordariomycetes(100);o__Hypocreales(100);f__Nectriaceae(100);g__Fusarium(94); |
| OTU8 | 70 | 953 | 694 | 422 | 212 | 109 | 191 | 214 | 279 | 5942 | 260 | 797 | 219 | 408 | 99 | 68 | 10937 | k__Fungi(100);p__Zygomycota(100);c__Mortierellomycotina_cls_Incertae_sedis(100);o__Mortierellales(100);f__Mortierellaceae(100);g__Mortierella(100);s__Mortierella_amoeboidea(76); |
| OTU6 | 20 | 12 | 242 | 121 | 0 | 0 | 0 | 0 | 2 | 10085 | 86 | 12 | 3 | 0 | 0 | 180 | 10763 | k__Fungi(100);p__Zygomycota(100);c__Mortierellomycotina_cls_Incertae_sedis(100);o__Mortierellales(100);f__Mortierellaceae(100);g__Mortierella(100);s__Mortierella_rishikesha(77); |
| OTU7 | 0 | 1 | 0 | 1 | 0 | 19 | 0 | 0 | 10 | 21 | 10553 | 0 | 0 | 0 | 5 | 0 | 10610 | k__Fungi(100);p__Basidiomycota(98);c__Microbotryomycetes(98);o__Sporidiobolales(98);f__Sporidiobolales_fam_Incertae_sedis(98);g__Rhodotorula(98);s__Rhodotorula_mucilaginosa(85); |
| OTU29 | 0 | 0 | 0 | 0 | 0 | 0 | 0 | 51 | 2 | 0 | 0 | 0 | 0 | 0 | 0 | 9126 | 9179 | k__Fungi(100);p__Basidiomycota(96);c__Agaricomycetes(96);o__Agaricales(96);f__Marasmiaceae(96);g__Marasmius(95); |
| OTU16 | 9 | 5239 | 10 | 176 | 0 | 47 | 41 | 34 | 33 | 115 | 1741 | 17 | 32 | 29 | 2 | 44 | 7569 | k__Fungi(100);p__Ascomycota(100);c__Saccharomycetes(97);o__Saccharomycetales(97);f__Saccharomycetales_fam_Incertae_sedis(93);g__Candida(93);s__Candida_xylopsoci(75); |
| OTU39 | 227 | 271 | 130 | 0 | 1 | 500 | 1348 | 1216 | 323 | 726 | 299 | 1088 | 1289 | 83 | 0 | 0 | 7501 | k__Fungi(100);p__Basidiomycota(92);c__Agaricomycetes(92);o__Cantharellales(92);f__Ceratobasidiaceae(92);g__Ceratobasidium(84);s__Ceratobasidium_sp(84); |
| OTU12 | 438 | 0 | 229 | 0 | 4516 | 1 | 191 | 2 | 0 | 0 | 19 | 1992 | 0 | 6 | 0 | 0 | 7394 | k__Fungi(100);p__Ascomycota(96);c__Sordariomycetes(73);o__Sordariales(61); |
| OTU30 | 0 | 484 | 1 | 446 | 1 | 0 | 691 | 2985 | 356 | 0 | 28 | 108 | 28 | 201 | 2056 | 0 | 7385 | k__Fungi(100);p__Ascomycota(90); |
| OTU17 | 1195 | 256 | 94 | 89 | 90 | 192 | 291 | 282 | 38 | 485 | 3706 | 100 | 185 | 156 | 84 | 51 | 7294 | k__Fungi(100);p__Ascomycota(100);c__Dothideomycetes(100);o__Pleosporales(100);f__Pleosporaceae(100);g__Alternaria(100); |
| OTU18 | 253 | 8 | 45 | 0 | 46 | 29 | 6 | 0 | 6306 | 0 | 98 | 62 | 0 | 0 | 4 | 15 | 6872 | k__Fungi(100);p__Ascomycota(100);c__Pezizomycotina_cls_Incertae_sedis(99);o__Pezizomycotina_ord_Incertae_sedis(99);f__Pezizomycotina_fam_Incertae_sedis(99);g__Knufia(99);s__Knufia_tsunedae(91); |
| OTU22 | 532 | 68 | 445 | 39 | 0 | 74 | 42 | 41 | 4485 | 314 | 348 | 53 | 8 | 27 | 0 | 155 | 6631 | k__Fungi(100);p__Ascomycota(100);c__Pezizomycotina_cls_Incertae_sedis(99);o__Pezizomycotina_ord_Incertae_sedis(99);f__Pezizomycotina_fam_Incertae_sedis(99);g__Knufia(99);s__Knufia_tsunedae(98); |
| OTU14 | 10 | 0 | 0 | 0 | 1 | 5 | 1100 | 0 | 45 | 78 | 0 | 527 | 4148 | 0 | 532 | 0 | 6446 | k__Fungi(100); |
| OTU33 | 55 | 0 | 194 | 269 | 627 | 152 | 85 | 67 | 0 | 213 | 0 | 200 | 212 | 6 | 0 | 4225 | 6305 | k__Fungi(100); |
| OTU25 | 4403 | 34 | 30 | 0 | 0 | 1250 | 94 | 14 | 181 | 0 | 30 | 0 | 2 | 1 | 0 | 146 | 6185 | k__Fungi(100);p__Ascomycota(100);c__Sordariomycetes(98);o__Sordariales(96);f__Lasiosphaeriaceae(96);g__Cercophora(71);s__Cercophora_mirabilis(71); |
| OTU23 | 0 | 1 | 0 | 500 | 131 | 0 | 630 | 1002 | 480 | 861 | 79 | 853 | 161 | 40 | 1389 | 0 | 6127 | k__Fungi(100);p__Ascomycota(91); |
| OTU19 | 0 | 6 | 37 | 1 | 0 | 1 | 2249 | 65 | 430 | 6 | 192 | 481 | 1918 | 14 | 625 | 0 | 6025 | k__Fungi(100); |
| OTU13 | 4708 | 18 | 0 | 0 | 0 | 1184 | 21 | 0 | 0 | 1 | 0 | 0 | 0 | 0 | 0 | 0 | 5932 | k__Fungi(100);p__Ascomycota(97); |
| OTU15 | 23 | 0 | 192 | 0 | 0 | 65 | 5512 | 36 | 0 | 0 | 0 | 0 | 0 | 0 | 0 | 0 | 5828 | k__Fungi(100);p__Ascomycota(95);c__Sordariomycetes(73);o__Sordariales(69); |
| OTU26 | 0 | 9 | 0 | 0 | 1 | 3 | 2335 | 106 | 228 | 0 | 123 | 40 | 1925 | 120 | 852 | 6 | 5748 | k__Fungi(100);p__Ascomycota(84);c__Lecanoromycetes(63); |
| OTU20 | 0 | 1 | 0 | 317 | 0 | 0 | 802 | 36 | 462 | 0 | 0 | 258 | 11 | 113 | 3734 | 0 | 5734 | k__Fungi(100); |
| OTU45 | 2624 | 383 | 416 | 104 | 80 | 365 | 372 | 56 | 253 | 124 | 62 | 187 | 89 | 246 | 7 | 9 | 5377 | k__Fungi(100);p__Ascomycota(100);c__Dothideomycetes(100);o__Pleosporales(100);f__Pleosporales_fam_Incertae_sedis(100); |
| OTU28 | 27 | 0 | 1 | 0 | 2 | 5161 | 0 | 0 | 1 | 0 | 0 | 0 | 0 | 2 | 0 | 0 | 5194 | k__Fungi(100); |
| OTU41 | 2 | 383 | 2785 | 33 | 0 | 21 | 55 | 239 | 0 | 18 | 0 | 5 | 0 | 0 | 0 | 1508 | 5049 | k__Fungi(100);p__Ascomycota(100);c__Eurotiomycetes(99);o__Eurotiales(99);f__Trichocomaceae(99);g__Aspergillus(99);s__Aspergillus_unilateralis(70); |
| OTU2197 | 40 | 3442 | 478 | 54 | 370 | 0 | 51 | 0 | 26 | 115 | 0 | 6 | 59 | 10 | 26 | 0 | 4677 | k__Fungi(100);p__Ascomycota(100);c__Sordariomycetes(99);o__Hypocreales(99);f__Nectriaceae(99);g__Fusarium(96); |
| OTU24 | 0 | 227 | 0 | 0 | 4379 | 0 | 0 | 0 | 0 | 0 | 0 | 5 | 0 | 3 | 8 | 0 | 4622 | k__Fungi(100);p__Ascomycota(64); |
| OTU32 | 104 | 21 | 0 | 0 | 81 | 0 | 2 | 0 | 0 | 0 | 4088 | 0 | 0 | 1 | 0 | 0 | 4297 | k__Fungi(100);p__Chytridiomycota(98);c__Chytridiomycetes(98);o__Rhizophlyctidales(98);f__Rhizophlyctidaceae(98);g__Rhizophlyctis(98);s__Rhizophlyctis_rosea(98); |
| OTU40 | 0 | 0 | 0 | 829 | 0 | 0 | 0 | 895 | 143 | 0 | 0 | 32 | 30 | 183 | 2115 | 0 | 4227 | k__Fungi(100); |
| OTU505 | 0 | 22 | 0 | 2 | 0 | 3 | 488 | 2 | 255 | 0 | 11 | 123 | 2663 | 0 | 633 | 0 | 4202 | k__Fungi(100);p__Ascomycota(90); |
| OTU38 | 0 | 268 | 9 | 18 | 1 | 15 | 5 | 0 | 0 | 0 | 3291 | 0 | 0 | 0 | 0 | 291 | 3898 | k__Fungi(100);p__Ascomycota(100);c__Eurotiomycetes(100);o__Eurotiales(100);f__Trichocomaceae(100);g__Talaromyces(100); |
| OTU27 | 3143 | 89 | 40 | 24 | 59 | 85 | 44 | 9 | 139 | 114 | 22 | 15 | 13 | 0 | 41 | 10 | 3847 | k__Fungi(100);p__Ascomycota(100);c__Dothideomycetes(100);o__Capnodiales(100);f__Davidiellaceae(91);g__Cladosporium(91);s__Cladosporium_delicatulum(73); |
| OTU37 | 0 | 54 | 0 | 0 | 0 | 0 | 0 | 0 | 0 | 0 | 3667 | 0 | 0 | 0 | 0 | 0 | 3721 | k__Fungi(100);p__Basidiomycota(100);c__Agaricomycetes(100);o__Agaricales(100);f__Agaricaceae(100);g__Leucoagaricus(100);s__Leucoagaricus_irinellus(100); |
| OTU80 | 0 | 0 | 0 | 3482 | 0 | 55 | 0 | 0 | 0 | 0 | 0 | 0 | 1 | 0 | 0 | 0 | 3538 | k__Fungi(100);p__Ascomycota(66); |
| OTU35 | 0 | 36 | 0 | 0 | 0 | 0 | 853 | 0 | 0 | 14 | 308 | 120 | 1 | 1460 | 671 | 1 | 3464 | k__Fungi(100);p__Ascomycota(96);c__unclassified_Ascomycota(75);o__unclassified_Ascomycota(75);f__unclassified_Ascomycota(75);g__unclassified_Ascomycota(75);s__Ascomycota_sp(75); |
| OTU42 | 2574 | 0 | 15 | 0 | 0 | 641 | 136 | 0 | 0 | 0 | 0 | 9 | 0 | 1 | 0 | 0 | 3376 | k__Fungi(100);p__Ascomycota(97); |
| OTU34 | 0 | 0 | 0 | 77 | 0 | 0 | 0 | 3249 | 0 | 0 | 0 | 0 | 0 | 0 | 25 | 0 | 3351 | k__Fungi(100);p__Ascomycota(90);c__Lecanoromycetes(62); |
| OTU31 | 3146 | 82 | 1 | 0 | 0 | 16 | 76 | 1 | 0 | 13 | 0 | 12 | 1 | 0 | 0 | 0 | 3348 | k__Fungi(100);p__Ascomycota(100);c__Dothideomycetes(97);o__Pleosporales(97);f__Pleosporales_fam_Incertae_sedis(67);g__Ascochyta(67);s__Ascochyta_manawaorae(67); |
| OTU70 | 0 | 0 | 1 | 0 | 0 | 0 | 0 | 0 | 0 | 3089 | 0 | 0 | 0 | 0 | 0 | 0 | 3090 | k__Fungi(100);p__Basidiomycota(84);c__Agaricomycetes(84);o__Agaricales(81); |
| OTU44 | 10 | 10 | 72 | 1025 | 0 | 0 | 6 | 191 | 258 | 494 | 0 | 565 | 90 | 13 | 182 | 0 | 2916 | k__Fungi(100);p__Ascomycota(92); |
| OTU36 | 0 | 0 | 0 | 897 | 1 | 8 | 0 | 0 | 299 | 1 | 0 | 0 | 335 | 1261 | 0 | 0 | 2802 | k__Fungi(100);p__Ascomycota(100);c__Sordariomycetes(100);o__Sordariales(100);f__Chaetomiaceae(100); |
| OTU58 | 11 | 18 | 182 | 0 | 0 | 139 | 0 | 15 | 4 | 0 | 0 | 2 | 0 | 0 | 0 | 2292 | 2663 | k__Fungi(100);p__Ascomycota(100);c__Dothideomycetes(88);o__Pleosporales(87);f__Pleosporales_fam_Incertae_sedis(82);g__Periconia(82);s__Periconia_prolifica(61); |
| OTU51 | 0 | 82 | 4 | 682 | 187 | 831 | 430 | 18 | 48 | 46 | 10 | 1 | 0 | 0 | 278 | 0 | 2617 | k__Fungi(100); |

Table S5. The information about top 50 OTUs soil Bacteria in soil samples.

| OTUId | Small tree | | | | | | | Shrub | | | | | | | Grass | | | | | | Bare patch | | | | | | 总计 | Taxonomy |
| --- | --- | --- | --- | --- | --- | --- | --- | --- | --- | --- | --- | --- | --- | --- | --- | --- | --- | --- | --- | --- | --- | --- | --- | --- | --- | --- | --- | --- |
|  | Plot 1 | | Plot 2 | Plot 3 | | Plot 4 | | Plot 1 | | Plot 2 | | Plot 3 | | Plot 4 | Plot 1 | Plot 2 | | Plot 3 | | Plot 4 | Plot 1 | Plot 2 | | Plot 3 | | Plot 4 |  |  |
| OTU1 | 3409 | 2417 | | | 2734 | | 2598 | 2690 | 2271 | | 2906 | | 4427 | | 2889 | | 3426 | 2898 | 2638 | | 6536 | 2112 | 2903 | | 4462 | | 51316 | sk__Bacteria(100);p__Proteobacteria(100);c__Gammaproteobacteria(100);o__Pseudomonadales(100);f__Pseudomonadaceae(100);g__Pseudomonas(100);s__Pseudomonas_azotoformans(100); |
| OTU2 | 319 | 675 | | | 1057 | | 437 | 837 | 814 | | 1556 | | 461 | | 190 | | 1022 | 1824 | 1075 | | 824 | 1569 | 492 | | 4 | | 13156 | sk__Bacteria(100);p__Acidobacteria(100);c__Blastocatellia(100);cnr__Blastocatellales(100);cnr__Blastocatellaceae_(Subgroup_4)(100);cnr__RB41(100);s__uncultured_Acidobacteriaceae_bacterium(73); |
| OTU39 | 189 | 487 | | | 485 | | 338 | 498 | 281 | | 444 | | 134 | | 175 | | 416 | 426 | 477 | | 295 | 258 | 262 | | 9 | | 5174 | sk__Bacteria(100);p__Acidobacteria(100);pnr__Subgroup_6(100);s__uncultured_bacterium(95); |
| OTU12 | 212 | 461 | | | 505 | | 358 | 408 | 221 | | 405 | | 189 | | 209 | | 336 | 443 | 505 | | 295 | 259 | 215 | | 67 | | 5088 | sk__Bacteria(100);p__Acidobacteria(100);pnr__Subgroup_6(100);s__uncultured_Saccharibacillus_sp.(61); |
| OTU9 | 220 | 368 | | | 558 | | 151 | 291 | 241 | | 415 | | 254 | | 34 | | 385 | 508 | 566 | | 558 | 392 | 112 | | 3 | | 5056 | sk__Bacteria(100);p__Acidobacteria(100);c__Blastocatellia(100);cnr__Blastocatellales(100);cnr__Blastocatellaceae_(Subgroup_4)(100);cnr__RB41(100);s__uncultured_Acidobacteria_bacterium(88); |
| OTU30 | 159 | 241 | | | 289 | | 134 | 309 | 298 | | 451 | | 335 | | 167 | | 496 | 432 | 142 | | 138 | 240 | 179 | | 251 | | 4261 | sk__Bacteria(100);p__Actinobacteria(100);c__Thermoleophilia(100);o__Solirubrobacterales(100);f__Solirubrobacteraceae(100);g__Solirubrobacter(100);s__uncultured_bacterium(77); |
| OTU3 | 186 | 128 | | | 253 | | 759 | 56 | 224 | | 103 | | 834 | | 543 | | 128 | 431 | 108 | | 73 | 151 | 171 | | 51 | | 4199 | sk__Bacteria(100);p__Acidobacteria(100);c__Blastocatellia(100);cnr__Blastocatellales(100);cnr__Blastocatellaceae_(Subgroup_4)(100);cnr__RB41(100);s__Acidobacterium_sp._Ac_12_G8(67); |
| OTU110 | 288 | 231 | | | 236 | | 202 | 412 | 372 | | 368 | | 210 | | 231 | | 449 | 359 | 236 | | 166 | 149 | 157 | | 13 | | 4079 | sk__Bacteria(100);p__Acidobacteria(100);pnr__Subgroup_6(100); |
| OTU7 | 146 | 211 | | | 288 | | 708 | 80 | 140 | | 150 | | 594 | | 125 | | 48 | 152 | 651 | | 50 | 228 | 153 | | 147 | | 3871 | sk__Bacteria(100);p__Acidobacteria(100);c__Blastocatellia(100);cnr__Blastocatellales(100);cnr__Blastocatellaceae_(Subgroup_4)(100);cnr__RB41(100); |
| OTU24 | 287 | 210 | | | 238 | | 123 | 163 | 212 | | 66 | | 121 | | 52 | | 61 | 436 | 424 | | 503 | 160 | 675 | | 0 | | 3731 | sk__Bacteria(100);p__Acidobacteria(100);c__Blastocatellia(100);cnr__Blastocatellales(100);cnr__Blastocatellaceae_(Subgroup_4)(100);cnr__RB41(100);s__uncultured_Acidobacteria_bacterium(90); |
| OTU21 | 74 | 492 | | | 137 | | 201 | 248 | 154 | | 622 | | 85 | | 45 | | 418 | 393 | 255 | | 137 | 127 | 172 | | 2 | | 3562 | sk__Bacteria(100);p__Proteobacteria(100);c__Betaproteobacteria(100);o__Nitrosomonadales(100);f__Nitrosomonadaceae(100);fnr__uncultured(100);s__uncultured_Burkholderiales_bacterium(89); |
| OTU18 | 82 | 126 | | | 194 | | 172 | 122 | 246 | | 433 | | 162 | | 55 | | 558 | 368 | 132 | | 134 | 254 | 147 | | 6 | | 3191 | sk__Bacteria(100);p__Actinobacteria(100);c__Thermoleophilia(100);o__Solirubrobacterales(100);onr__288-2(89);s__uncultured_bacterium(88); |
| OTU27 | 138 | 253 | | | 264 | | 77 | 396 | 215 | | 194 | | 267 | | 105 | | 231 | 165 | 145 | | 150 | 125 | 241 | | 28 | | 2994 | sk__Bacteria(100);p__Chloroflexi(100);pnr__KD4-96(100);s__uncultured_Chloroflexi_bacterium(85); |
| OTU6 | 24 | 234 | | | 69 | | 133 | 31 | 81 | | 27 | | 592 | | 41 | | 107 | 24 | 29 | | 20 | 38 | 11 | | 1525 | | 2986 | sk__Bacteria(100);p__Actinobacteria(100);c__Actinobacteria(100);o__Pseudonocardiales(100);f__Pseudonocardiaceae(100);g__Crossiella(100); |
| OTU5 | 18 | 129 | | | 361 | | 72 | 366 | 241 | | 323 | | 63 | | 5 | | 258 | 279 | 297 | | 303 | 90 | 115 | | 0 | | 2920 | sk__Bacteria(100);p__Gemmatimonadetes(100);c__Gemmatimonadetes(100);o__Gemmatimonadales(100);f__Gemmatimonadaceae(100);fnr__uncultured(86);s__uncultured_Gemmatimonas_sp.(76); |
| OTU8 | 95 | 182 | | | 117 | | 87 | 180 | 129 | | 234 | | 159 | | 116 | | 255 | 196 | 237 | | 158 | 145 | 137 | | 115 | | 2542 | sk__Bacteria(100);p__Actinobacteria(100);c__Acidimicrobiia(100);o__Acidimicrobiales(100);onr__uncultured(100);s__uncultured_Acidothermaceae_bacterium(73); |
| OTU92 | 171 | 166 | | | 82 | | 330 | 86 | 159 | | 175 | | 194 | | 349 | | 183 | 234 | 135 | | 69 | 65 | 70 | | 61 | | 2529 | sk__Bacteria(100);p__Acidobacteria(100);c__Holophagae(100);cnr__Subgroup_7(100);s__uncultured_Acidobacterium_sp.(92); |
| OTU35 | 145 | 134 | | | 148 | | 144 | 180 | 180 | | 263 | | 153 | | 189 | | 249 | 215 | 99 | | 82 | 120 | 163 | | 6 | | 2470 | sk__Bacteria(100);p__Gemmatimonadetes(100);c__Gemmatimonadetes(100);o__Gemmatimonadales(100);f__Gemmatimonadaceae(100);fnr__uncultured(100);s__uncultured_Gemmatimonadales_bacterium(100); |
| OTU2830 | 106 | 120 | | | 285 | | 341 | 175 | 480 | | 77 | | 53 | | 216 | | 41 | 148 | 114 | | 66 | 100 | 12 | | 1 | | 2335 | sk__Bacteria(100);p__Acidobacteria(100);c__Blastocatellia(100);cnr__Blastocatellales(100);cnr__Blastocatellaceae_(Subgroup_4)(100);cnr__RB41(100);s__uncultured_bacterium(96); |
| OTU114 | 228 | 118 | | | 194 | | 238 | 154 | 271 | | 134 | | 100 | | 249 | | 112 | 174 | 127 | | 123 | 88 | 17 | | 1 | | 2328 | sk__Bacteria(100);p__Acidobacteria(100);c__Blastocatellia(100);cnr__Blastocatellales(100);cnr__Blastocatellaceae_(Subgroup_4)(100);cnr__RB41(100);s__uncultured_bacterium(66); |
| OTU26 | 299 | 73 | | | 133 | | 121 | 75 | 85 | | 88 | | 263 | | 335 | | 94 | 71 | 103 | | 91 | 12 | 225 | | 208 | | 2276 | sk__Bacteria(100);p__Proteobacteria(100);c__Betaproteobacteria(100);o__Nitrosomonadales(100);f__Nitrosomonadaceae(100);fnr__uncultured(100);s__uncultured_Oxalobacteraceae_bacterium(85); |
| OTU41 | 62 | 243 | | | 108 | | 138 | 89 | 106 | | 208 | | 146 | | 58 | | 174 | 215 | 251 | | 83 | 198 | 105 | | 64 | | 2248 | sk__Bacteria(100);p__Proteobacteria(100);c__Alphaproteobacteria(100);o__Rhizobiales(100);f__Xanthobacteraceae(82); |
| OTU33 | 103 | 120 | | | 244 | | 203 | 107 | 100 | | 119 | | 232 | | 102 | | 136 | 163 | 61 | | 63 | 24 | 228 | | 77 | | 2082 | sk__Bacteria(100);p__Proteobacteria(100);c__Betaproteobacteria(100);o__Nitrosomonadales(100);f__Nitrosomonadaceae(100);fnr__uncultured(100);s__uncultured_Burkholderiaceae_bacterium(81); |
| OTU40 | 120 | 97 | | | 89 | | 196 | 58 | 120 | | 119 | | 192 | | 353 | | 97 | 167 | 178 | | 79 | 74 | 65 | | 45 | | 2049 | sk__Bacteria(100);p__Acidobacteria(100);c__Holophagae(100);cnr__Subgroup_7(100); |
| OTU31 | 24 | 81 | | | 257 | | 138 | 72 | 173 | | 370 | | 85 | | 12 | | 274 | 147 | 85 | | 101 | 166 | 15 | | 1 | | 2001 | sk__Bacteria(100);p__Actinobacteria(100);c__Rubrobacteria(99);o__Rubrobacterales(99);onr__Rubrobacteriaceae(99);g__Rubrobacter(99);s__uncultured_Actinomycetales_bacterium(99); |
| OTU62 | 69 | 127 | | | 124 | | 86 | 210 | 88 | | 211 | | 131 | | 76 | | 139 | 141 | 136 | | 65 | 131 | 169 | | 61 | | 1964 | sk__Bacteria(100);p__Acidobacteria(100);pnr__Subgroup_6(100);s__uncultured_bacterium(71); |
| OTU2131 | 5 | 209 | | | 86 | | 55 | 251 | 56 | | 394 | | 5 | | 9 | | 272 | 255 | 90 | | 35 | 76 | 12 | | 0 | | 1810 | sk__Bacteria(100);p__Gemmatimonadetes(100);c__Gemmatimonadetes(100);o__Gemmatimonadales(100);f__Gemmatimonadaceae(100);fnr__uncultured(89);s__uncultured_Gemmatimonas_sp.(80); |
| OTU14 | 68 | 45 | | | 77 | | 76 | 44 | 52 | | 186 | | 101 | | 41 | | 128 | 163 | 39 | | 29 | 116 | 93 | | 500 | | 1758 | sk__Bacteria(100);p__Actinobacteria(100);c__Actinobacteria(100);o__Corynebacteriales(100);f__Mycobacteriaceae(100);g__Mycobacterium(100); |
| OTU4 | 118 | 72 | | | 99 | | 87 | 88 | 69 | | 114 | | 133 | | 107 | | 124 | 106 | 84 | | 212 | 102 | 117 | | 125 | | 1757 | sk__Bacteria(100);p__Proteobacteria(100);c__Gammaproteobacteria(100);o__Enterobacteriales(100);f__Enterobacteriaceae(100);fnr__Escherichia-Shigella(100);g__Escherichia(100);s__Escherichia_coli(100);Escherichia_coli_K-12(100); |
| OTU102 | 331 | 38 | | | 97 | | 140 | 47 | 157 | | 58 | | 164 | | 343 | | 78 | 54 | 28 | | 44 | 12 | 89 | | 40 | | 1720 | sk__Bacteria(100);p__Proteobacteria(100);c__Betaproteobacteria(100);o__Burkholderiales(100);f__Comamonadaceae(100);g__Ramlibacter(98);s__uncultured_bacterium(86); |
| OTU574 | 98 | 115 | | | 103 | | 102 | 98 | 61 | | 152 | | 139 | | 40 | | 77 | 136 | 79 | | 92 | 79 | 197 | | 117 | | 1685 | sk__Bacteria(100);p__Acidobacteria(100);pnr__Subgroup_6(100); |
| OTU13 | 65 | 114 | | | 52 | | 127 | 153 | 61 | | 248 | | 4 | | 10 | | 123 | 216 | 116 | | 133 | 173 | 77 | | 0 | | 1672 | sk__Bacteria(100);p__Nitrospirae(100);c__Nitrospira(100);o__Nitrospirales(100);onr__0319-6A21(100);s__uncultured_bacterium(66); |
| OTU156 | 112 | 84 | | | 119 | | 44 | 92 | 95 | | 188 | | 234 | | 78 | | 89 | 78 | 46 | | 58 | 53 | 89 | | 163 | | 1622 | sk__Bacteria(100);p__Actinobacteria(100);c__Actinobacteria(100);o__Micromonosporales(100);f__Micromonosporaceae(100);g__Phytohabitans(68);s__uncultured_bacterium(68); |
| OTU95 | 102 | 112 | | | 102 | | 246 | 84 | 114 | | 63 | | 136 | | 139 | | 56 | 116 | 123 | | 47 | 45 | 79 | | 50 | | 1614 | sk__Bacteria(100);p__Proteobacteria(100);c__Betaproteobacteria(100);cnr__TRA3-20(100);s__uncultured_beta_proteobacterium(91); |
| OTU152 | 27 | 98 | | | 144 | | 45 | 114 | 148 | | 241 | | 46 | | 17 | | 202 | 222 | 72 | | 76 | 93 | 11 | | 2 | | 1558 | sk__Bacteria(100);p__Actinobacteria(100);c__Thermoleophilia(100);o__Gaiellales(100);f__Gaiellaceae(100);g__Gaiella(100);s__uncultured_bacterium(100); |
| OTU20 | 116 | 85 | | | 80 | | 39 | 116 | 80 | | 416 | | 108 | | 88 | | 71 | 63 | 36 | | 20 | 76 | 142 | | 4 | | 1540 | sk__Bacteria(100);p__Proteobacteria(100);c__Gammaproteobacteria(100);o__Xanthomonadales(100);onr__Xanthomonadales_Incertae_Sedis(100);g__Steroidobacter(100);s__uncultured_bacterium(64); |
| OTU22 | 159 | 154 | | | 97 | | 209 | 78 | 100 | | 59 | | 61 | | 254 | | 149 | 105 | 28 | | 29 | 21 | 8 | | 19 | | 1530 | sk__Bacteria(100);p__Proteobacteria(100);c__Alphaproteobacteria(100);o__Rhizobiales(100);onr__JG34-KF-361(100);s__uncultured_bacterium(90); |
| OTU2420 | 66 | 98 | | | 120 | | 319 | 14 | 77 | | 50 | | 197 | | 226 | | 43 | 57 | 77 | | 34 | 68 | 74 | | 8 | | 1528 | sk__Bacteria(100);p__Acidobacteria(100);c__Blastocatellia(100);cnr__Blastocatellales(100);cnr__Blastocatellaceae_(Subgroup_4)(100);cnr__RB41(100);s__uncultured_Acidobacterium_sp.(65); |
| OTU15 | 41 | 28 | | | 67 | | 32 | 47 | 56 | | 142 | | 190 | | 23 | | 66 | 73 | 22 | | 25 | 91 | 107 | | 507 | | 1517 | sk__Bacteria(100);p__Proteobacteria(100);c__Alphaproteobacteria(100);o__Rhizobiales(100);f__Bradyrhizobiaceae(100);g__Bradyrhizobium(99); |
| OTU77 | 51 | 102 | | | 105 | | 59 | 184 | 50 | | 221 | | 23 | | 27 | | 94 | 118 | 187 | | 95 | 134 | 60 | | 0 | | 1510 | sk__Bacteria(100);p__Proteobacteria(100);c__Deltaproteobacteria(100);o__Desulfurellales(100);f__Desulfurellaceae(100);fnr__H16(100);s__uncultured_Acidobacteriales_bacterium(66); |
| OTU52 | 97 | 129 | | | 105 | | 101 | 133 | 52 | | 64 | | 107 | | 148 | | 144 | 92 | 53 | | 61 | 47 | 111 | | 30 | | 1474 | sk__Bacteria(100);p__Acidobacteria(100);pnr__Subgroup_6(100);s__uncultured_Acidobacterium_sp.(90); |
| OTU979 | 51 | 114 | | | 135 | | 72 | 65 | 144 | | 131 | | 50 | | 19 | | 150 | 161 | 62 | | 155 | 54 | 10 | | 2 | | 1375 | sk__Bacteria(100);p__Actinobacteria(100);c__Thermoleophilia(100);o__Gaiellales(100);onr__uncultured(89);s__uncultured_bacterium(73); |
| OTU10 | 32 | 73 | | | 66 | | 43 | 464 | 44 | | 118 | | 35 | | 4 | | 71 | 87 | 146 | | 100 | 43 | 13 | | 0 | | 1339 | sk__Bacteria(100);p__Chloroflexi(100);pnr__P2-11E(100);s__uncultured_bacterium(100); |
| OTU54 | 119 | 114 | | | 94 | | 56 | 177 | 41 | | 162 | | 73 | | 82 | | 116 | 71 | 78 | | 46 | 46 | 41 | | 7 | | 1323 | sk__Bacteria(100);p__Acidobacteria(100);pnr__Subgroup_6(100);s__Acidobacteria_bacterium_CB_286306(73); |
| OTU72 | 68 | 121 | | | 46 | | 108 | 25 | 48 | | 140 | | 12 | | 48 | | 128 | 312 | 88 | | 76 | 73 | 22 | | 0 | | 1315 | sk__Bacteria(100);p__Proteobacteria(100);c__Betaproteobacteria(100);o__Nitrosomonadales(100);f__Nitrosomonadaceae(100);fnr__uncultured(100);s__uncultured_Burkholderiaceae_bacterium(74); |
| OTU28 | 38 | 51 | | | 58 | | 18 | 99 | 28 | | 345 | | 58 | | 16 | | 110 | 155 | 65 | | 26 | 126 | 105 | | 0 | | 1298 | sk__Bacteria(100);p__Proteobacteria(100);c__Alphaproteobacteria(100);o__Rhodospirillales(100);f__Rhodospirillaceae(100);fnr__uncultured(100);s__uncultured_Dongia_sp.(96); |
| OTU262 | 60 | 110 | | | 78 | | 73 | 113 | 63 | | 84 | | 107 | | 39 | | 77 | 80 | 68 | | 58 | 78 | 146 | | 59 | | 1293 | sk__Bacteria(100);p__Proteobacteria(100);c__Alphaproteobacteria(100);o__Rhizobiales(100);f__Xanthobacteraceae(92); |
| OTU66 | 86 | 87 | | | 46 | | 163 | 69 | 80 | | 117 | | 23 | | 87 | | 92 | 247 | 56 | | 33 | 32 | 44 | | 0 | | 1262 | sk__Bacteria(100);p__Proteobacteria(100);c__Betaproteobacteria(100);o__Nitrosomonadales(100);f__Nitrosomonadaceae(100);fnr__uncultured(100);s__uncultured_Burkholderiales_bacterium(75); |
| OTU333 | 269 | 92 | | | 35 | | 97 | 43 | 59 | | 123 | | 18 | | 89 | | 67 | 37 | 155 | | 80 | 41 | 43 | | 2 | | 1250 | sk__Bacteria(100);p__Acidobacteria(100);c__Holophagae(100);cnr__Subgroup_10(100);cnr__ABS-19(100);s__uncultured_soil_bacterium(84); |
| OTU139 | 19 | 59 | | | 207 | | 416 | 12 | 48 | | 45 | | 143 | | 30 | | 21 | 51 | 82 | | 6 | 75 | 23 | | 2 | | 1239 | sk__Bacteria(100);p__Acidobacteria(100);c__Blastocatellia(100);cnr__Blastocatellales(100);cnr__Blastocatellaceae_(Subgroup_4)(100);cnr__RB41(100); |
